# PEARL-Neuro Database: EEG, fMRI, health and lifestyle data of middle-aged people at risk of dementia

**DOI:** 10.1038/s41597-024-03106-5

**Published:** 2024-03-07

**Authors:** Patrycja Dzianok, Ewa Kublik

**Affiliations:** Laboratory of Emotions Neurobiology, Nencki Institute of Experimental Biology PAS, Warsaw, Poland

**Keywords:** Risk factors, Cognitive ageing, Alzheimer's disease

## Abstract

Interdisciplinary approaches are needed to understand the relationship between genetic factors and brain structure and function. Here we describe a database that includes genetic data on apolipoprotein E (*APOE*) and phosphatidylinositol binding clathrin assembly protein (*PICALM*) genes, both of which are known to increase the risk of late-onset Alzheimer's disease, paired with psychometric (memory, intelligence, mood, personality, stress coping strategies), basic demographic and health data on a cohort of 192 healthy middle-aged (50–63) individuals. Part of the database (~79 participants) also includes blood tests (blood counts, lipid profile, HSV virus) and functional neuroimaging data (EEG/fMRI) recorded with a resting-state protocol (eyes open and eyes closed) and two cognitive tasks (multi-source interference task, MSIT; and Sternberg's memory task). The data were validated and showed overall good quality. This open-science dataset is well suited not only for research relating to susceptibility to Alzheimer's disease but also for more general questions on brain aging or can be used as part of meta-analytical multi-disciplinary projects.

## Background & Summary

We describe the Polish Electroencephalography, Alzheimer's Risk-genes, Lifestyle and Neuroimaging (PEARL-Neuro) Database, collected from 192 middle-aged (50-63) participants, which includes following data: genetic information on two genes polymorphisms (*APOE/PICALM* variants) along with basic demography, family history of dementia, results of seven psychometric tests and (in a subset of 79 participants) neuroimaging data (including EEG and fMRI data), and basic blood tests. The data was collected in line with the growing imaging genomics movement, a field that integrates genotyping with multiple imaging technologies to understand the complex relationship between genetics and the functioning of the organism. The approach is increasingly used in neuroscience to link genes to brain functions^1–3^. By identifying genetic variations that are associated with brain anatomical and/or functional phenotypes, imaging genomics indicates putative biomarkers that can be used to predict the risk of developing neurological and psychiatric disorders. This can lead to an earlier diagnosis, more targeted treatments, and better outcomes for patients.

Alzheimer's disease (AD) is a progressive neurological disorder that affects a person’s cognitive abilities, including memory, language, perception, and decision-making. Genome-wide association studies (GWAS) have identified many genetic variants that are associated with the development of AD. However, in the case of sporadic, late-onset AD (LOAD), there is no known causative gene; all identified genes only modify the risk. The most commonly identified risk gene is for apolipoprotein E (*APOE*), and another is for phosphatidylinositol binding clathrin assembly protein (*PICALM*)^4–6^. The *APOE* gene has three common variants: ε2, ε3, and ε4, with ε4 being related to an increased risk of developing LOAD. The number of *APOE* associated mechanisms causing AD is currently under investigation, including metabolism and clearance of amyloid-β (amyloid plaques are a hallmark of AD), tau pathology (neurofibrillary tangles are also a hallmark of AD), inflammation, and oxidative stress and other^7^. *PICALM* is involved in the endocytosis process and is also linked to disturbances in amyloid-β aggregation/clearance. Genetic studies have identified multiple variations of the *PICALM* gene that are associated with an increased risk of developing AD. Specifically, an rs3851179 variant was linked to AD risk. *APOE* and *PICALM* have also been linked to other disorders. The ε4 allele has been reported to increase the risk of cardiovascular disease, including coronary artery disease, stroke, and atherosclerosis, which, in turn, can also influence brain health.

Given that AD is a complex and multifactorial disease with many overlapping etiological factors that can interact with each other and contribute to disease development, a broad research approach is needed. Therefore, in addition to genotyping, our database includes neuroimaging data, along with basic demographic and health information. In terms of functional brain imaging, we included electroencephalography (EEG) and functional magnetic resonance imaging (fMRI). The two methods complement each other — EEG provides good temporal resolution while fMRI provides good spatial resolution.

An important issue in AD is its unknown onset time and early stages of progression. Evident symptoms are diagnosed too late, when the brain damage is substantial and irreversible. It is necessary to learn how to detect earlier signs of impending disease. Therefore, to build the database, we invited participants between the age of 50 and 63 (55.05 ± 3.09 years old), i.e., the decade immediately preceding the contractual limit for the onset of the disease. The main goal of the study was to check if, in this population, people carrying risk alleles are already showing the beginnings of AD-typical symptoms, which we briefly describe in the following paragraphs.

EEG is a non-invasive method of measuring the electrical activity of the brain. The most visible functional hallmark among AD patients is the so-called “slowing of EEG,” which corresponds to a shift in the brain waves’ power spectrum to slower frequencies^8^. This EEG hallmark, along with decreased signal complexity, is thought to reflect neuronal degeneration and disturbances within the cholinergic system. EEG has also been used to investigate changes in brain activity that occur during specific cognitive tasks, such as memory tasks. Studies have shown that individuals with AD have reduced EEG activity in the regions of the brain that are involved in memory processing compared to healthy individuals. MRI has been used to investigate changes in brain structure in AD patients. One of the key structural changes that occur in AD is the loss of brain tissue, particularly in the hippocampus and other medial temporal lobe structures that are involved in memory processing^9^. fMRI, on the other hand, can help to detect differences in the activation of specific brain regions during cognitive tasks by measuring changes in blood flow. One of the key changes that occurs in AD is the disruption of neural networks in the brain, including the default mode network (DMN)^10^.

Psychometric testing in AD patients has indicated that personality traits that may accompany the disease and/or may predispose to its development include depression and anxiety, mood changes, agitation and aggression, apathy, neuroticism, and impulsivity^6,11^.

Blood sampling is a relatively cheap and routine diagnostic approach to test the health condition of an organism/multiple body organs. *APOE* is implicated in lipid metabolism, and, indeed, biochemical blood tests have provided evidence that high levels of cholesterol may be a risk factor for the development of AD^12^. Other studies have found that people with AD, and also subjects with a greater risk for the disease, may have changes in their blood counts (i.e., numbers and proportions of blood cells)^13,14^. Changes within granulocyte profiles were found in patients^15,16^ and in people at risk of AD^14,17^ which is in line with data suggesting that chronic (neuro)inflammation can be a significant factor in the development and progression of AD^18^. Additionally, some researchers have suggested that herpes simplex virus (HSV) infection may be linked to AD by promoting inflammation and damage in the brain^19^. There are reports that people with AD may be more likely to have a history of HSV infection compared to people without the condition, but the relationship between the two is complex and not yet fully understood^20^.

Overall, imaging genomics is an important tool for understanding the complex genetic and neurobiological factors that contribute to AD and other dementias. Combined with other health and psychometric tests (as in our database), data can be analyzed with multivariate approaches and holds the potential to identify patterns of symptoms estimating AD risk, indicate new targets for therapy, and help guide the development of personalized treatment strategies for individuals with the disease.

Our goal while collecting the data was focused on AD, but the dataset can be used to answer other questions within both neurocognitive and clinical fields. In particular, the studies focused on age-related aspects of brain activity or psychology/cognitive aspects (psychometric tests) can benefit from the dataset. It may also be of interest for research on cardiovascular diseases, as the APOE gene has been implicated as a risk factor. The dataset is large enough to support individual analyses and can be utilized as part of meta-analytical, multi-disciplinary projects.

Currently, we are not aware of any similar publicly released dataset. There is a growing number of studies collecting and analyzing neuroimaging or health related data from matched populations of early stage AD or healthy participants exhibiting selected markers of Alzheimer's disease (both genetic or derived from biosamples; and both well-established or newly described biomarkers)^14,21–28^. Most of them concern elderly or young individuals, with fewer data related to middle-aged participants. Moreover, the data associated with these individual studies are not openly shared (to the authors best knowledge during the time of releasing our dataset). There are collections related to Alzheimer's Disease, among which The Alzheimer's Disease Neuroimaging Initiative (ADNI) database^29^ stands out as the largest (also including information about elderly controls). It contains basic demographic information, MRI and positron emission tomography (PET) images, selected genetic factors, simple AD-related cognitive tests, and blood tests results. Although, no functional data is available through ADNI database, and it is only partially open. Access is restricted to registered users who must obtain individual approval to use the data and are required to submit yearly use reports. National Alzheimer's Coordinating Center (NACC) also collects and maintains data related to Alzheimer's disease from multiple research projects and studies across the United States (submitting a data request is necessary)^30^. The other example is The Alzheimer's Disease Data Initiative (ADDI), which aims to accelerate AD research by providing an open-access platform for sharing data (includes various types of datasets with clinical, imaging, and genetic information)^31^.

We share this dataset as part of the open-data movement, a global initiative aimed at promoting the sharing of data and information in a transparent and accessible manner. There is a huge need to make neuroimaging and health/medical databases available to the wider scientific community, which is the first step in overcoming the current reproducibility crisis and may help to improve future research. This need is especially visible in computational neuroscience^32,33^. The data are prepared in machine-readable BIDS format, so they can be easily analyzed. Additionally, the database is stored at the OpenNeuro repository^34^, which makes it possible to gain access easily with DataLad, a data management solution, or directly from the OpenNeuro website^35^.

## Methods

### Ethics

This study was conducted in accordance with the declaration of Helsinki and was approved by the Bioethics Committee of the Nicolaus Copernicus University in Toruń at the Ludwik Rydygier Collegium Medicum in Bydgoszcz, Poland (KB 684/2019). All participants (N = 200) provided written informed consent and signed the extended note regarding study information, including information about data privacy and its pseudo-anonymization and anonymization for the purpose of analyses and publications related to the research project. The public database is now fully anonymized. After signing the informed consent, participants received their own results regarding genetic screening and psychometric scores. To avoid a bias in consecutive stages of the study, the results were handed out after the final experimental session. Participants included in the second stage of the study also received a cash remuneration (100 PLN, ~21 EUR) and their own MRI results. Additionally, we asked participants to declare if they wanted to receive the results of genetic tests (and other tests as well). Out of the 200 participants taking part in the study, 192 signed an addendum, agreeing to make the research data publicly available in the open scientific database – these data is described here.

### General information

**Fig. 1:**
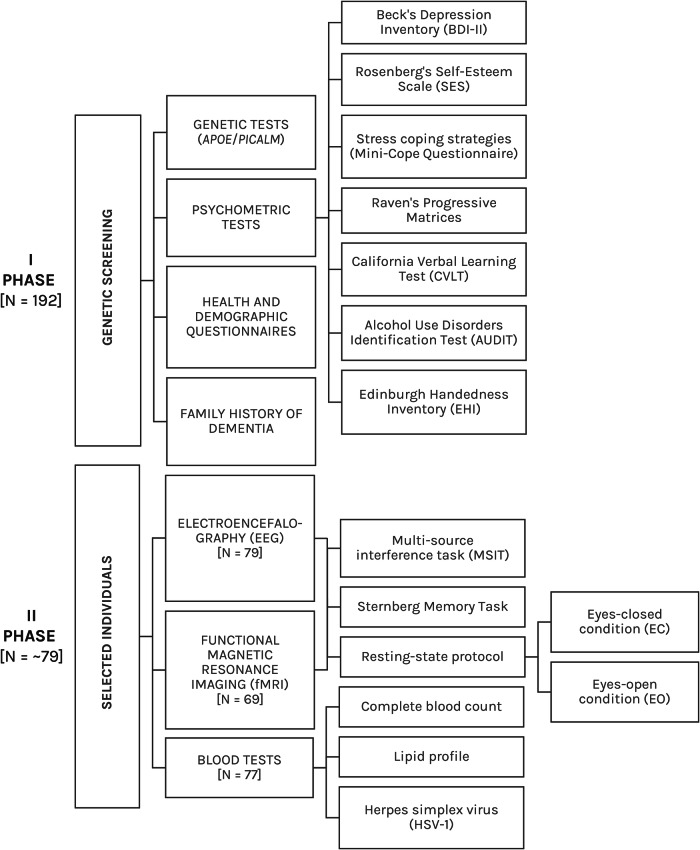
Diagram of the study methodology, which illustrates both study phases and conducted experiments. *The exact number of participants changed at different stages of the study. See the Missing data section for more information.

**Fig. 2:**
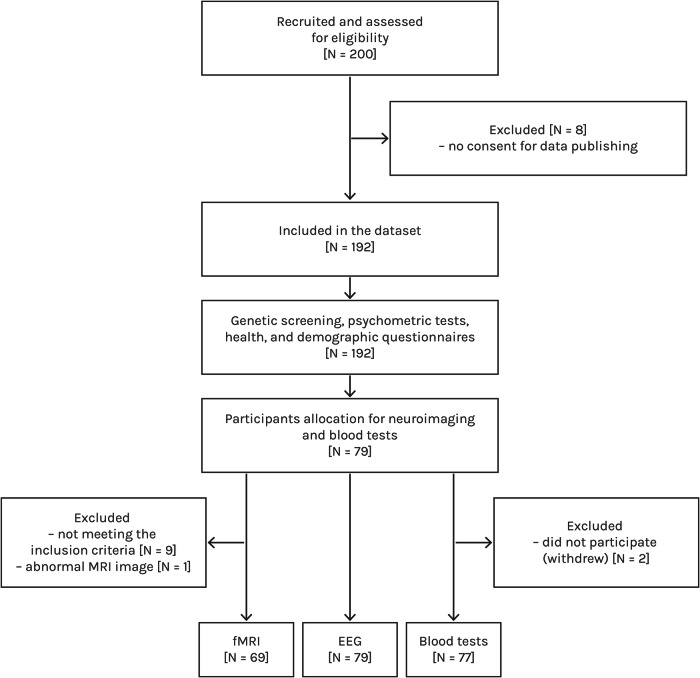
Flow diagram illustrating the study design, including participant selection and the quantity of data included within this open dataset.

One hundred ninety two middle-aged individuals (50–63 years old) completed the first phase of experiments, and 79 participants continued the experiment through its second phase (Figs 1, 2, Table 1). The first phase of the experiment included genetic screening (only *APOE/PICALM* polymorphisms) and a meeting during which participants completed psychometric tests (see Fig. 1 and the next paragraph for a list of tests) and general, extensive questionnaires on health and demographic data. In the second phase of the experiment, subjects with risk alleles for *APOE/PICALM* genes and a matched group of subjects with neutral alleles were invited to participate in the neuroimaging sessions. EEG recordings and fMRI scans were performed on separate days. The EEG session was conducted in the EEG laboratory at the Nencki Institute of Experimental Biology PAS, and the fMRI session was conducted in the Bioimaging Research Center at the Institute of Physiology and Pathology of Hearing in Poland. An approximately equal number of subjects underwent an EEG session, followed by an fMRI session, and vice versa to avoid a task learning bias in either design (N = 31 EEG session was administered first, N = 39 fMRI session was administered first; the order is indicated in the database). During both sessions, we used a resting-state protocol, as well as two cognitive tasks, one examining broad executive functions (Multi-Source Cognitive Task, MSIT) and the other examining memory (Sternberg Memory Task). Blood samples for complete blood count (CBC), lipid profile and antibodies against herpes simplex virus (HSV-1) were collected on a separate day.

**Table 1:** Blood test laboratory norms.

Parameter	Laboratory norm [K/μl]	
	Females	Males
Leukocytes [K/µl]	3.98–10.4	4.23–9.07
Erythrocytes [K/µl]	3.93–5.22	4.63–6.08
Hemoglobin [K/µl]	11.20–15.70	13.70–17.50
Hematocrit [%]	34.10–44.90	40.10–51.0
MCV [fl]	79.40–94.80	79–92.20
MCH [pg]	25.60–32.20	25.70–32.20
MCHC [g/dl]	32.20–35.50	32.30–36.50
RDW-CV [%]	11.70–14.40	11.60–14.40
Platelets [K/µl]	150–400	
PDW [fl]	9.80–16.20	9.80–16.10
MPV [fl]	9.40–12.50	9.40–12.60
P-LCR [%]	19.10–46.60	19.20–47
Neutrophils [K/µl | %]	2–7 (40–80%)	
Lymphocytes [K/µl | %]	1–3 (20–40%)	
Monocytes [K/µl | %]	0.2–1 (2–10%)	
Eosinophils [K/µl | %]	0.02–0.5 (1–6%)	
Basophils [K/µl | %]	0.02–0.10 (0–2%)	
Total cholesterol [mg/dl]	115–190	
HDL cholesterol [mg/dl]	> = 45	> = 40
Non-HDL cholesterol [mg/dl]	<145*	
LDL cholesterol [mg/dl]	<115*	
Triglycerides [mg/dl]	max. 150	

*for healthy people at low or intermediate risk of death from cardiovascular disease.

The subjects were asked to stop taking medications 24 hours before the EEG study unless it was necessary in relation to a chronic condition. Subjects were also asked to refrain from consuming alcohol, stimulating and caffeinated beverages, and psychoactive substances 24 hours before the study. However, if it was natural for the subject to drink a cup of coffee in the morning, we asked them to maintain their natural daily rhythm. Subjects were asked to be well-rested before either session. The exclusion criteria for the study were as follows (the participants were asked about the exclusions during an initial online survey, some of the questions were additionally confirmed in the survey before fMRI sessions, and all of them were printed on the “Information about the study” sheet that was given to the participants before the first EEG or fMRI session for signing):

- recent or ongoing infection,
- general excessive health problems,
- epilepsy,
- known mental illness or brain damage,
- chronic headaches,
- sleep disorders,
- skin diseases,
- metal objects/implants in the body,
- pregnancy.

### Demographic, health and psychometric questionnaires

The demographic and health questionnaire included the following information:

- Demographic data: age, sex, education level.
- Health data: learning difficulties (dyslexia, etc.), body-mass index (BMI), diabetes, hypertension, thyroid diseases, other chronic diseases, allergies, permanent drug intake, nonsteroidal anti-inflammatory drugs intake, smoking status, caffeine intake, alcohol consumption (measured by AUDIT test).
- Blood tests: leukocytes, erythrocytes, hemoglobin, hematocrit, MCV, MCH, MCHC, RDW-CV, platelets, PDW, MPV, P-LCR, neutrophils, lymphocytes, monocytes, eosinophils, basophils, total cholesterol, HDL cholesterol, non-HDL cholesterol, LDL cholesterol, triglycerides, HSV.
- Additional data: session order (EEG/fMRI).
- Genetic data: *APOE* haplotype, *PICALM* rs3851179 genotype.
- Family history of dementia data (parents).

Participants filled in a battery of psychometric tests (Fig. 1), including:

- Beck’s Depression Inventory (BDI): measured depression and mood changes. This widely used test consisted of 21 items that assessed various symptoms of depression/mood, such as sadness, guilt, fatigue or loss of interest. The total score ranged from 0 to 63. The database includes the final score for each participant^36,37^.
- Rosenberg’s Self-Esteem Scale (SES): measured self-esteem and confidence. The test consisted of 10 statements that assessed both positive and negative feelings about oneself. Scores ranged from 0 to 30 points. The database includes the final score for each participant^38,39^.
- Mini-Cope Questionnaire: measured stress-coping strategies used in daily life situations. The test consisted of 28 items. The database includes 14 basic items that could be further recalculated^40,41^.
- NEO-FFI Personality Inventory: measured the personality traits known as the Big Five personality traits. The scale measured: neuroticism, extraversion, openness to experience, agreeableness, and conscientiousness. The test consisted of 60 items. The database includes the final score for each measure^42–44^.
- Raven’s Progressive Matrices (RPM) in standard/classic version: measured fluid intelligence and the ability to solve complex, novel tasks. We changed the time that the test subjects had to solve the test to 30 minutes (from unlimited time in the classic version)^45,46^.
- California Verbal Learning Test (CVLT): measured memory abilities and verbal learning. The CVLT provided several measures, including total recall, recall by trials, recognition memory, and others. The test was ecological, meaning that the questions were related to daily activities, like memorizing shopping lists^47,48^.

Handedness inventory (all participants were right-handed)^49^.

All psychological tests were the Polish adaptation and standardized versions and were obtained from the Psychological Test Laboratory of the Polish Psychological Association. The health information included in the ‘demographic & health’ questionnaire was provided by the participants, so the answers were subjective and not medically verified (i.e., hypertension or diabetes).

### Blood tests

Blood testing was outsourced to a third-party, certified medical laboratory facility. Samples were collected by a trained nurse in the morning at the local facility. HSV tests were performed using the ELISA method (Euroimmun kits), testing the IgG antibodies. The blood test laboratory norms for the performed tests are shown in Table 1.

### Genetic screening

Genetic screening was outsourced to a third-party company (Genomed S.A., Poland). Buccal swab samples were collected by brushing a swab on the surface of the inner cheek (buccal mucosa) (we used COPAN eNat® buccal swabs especially designed for nucleic acid collection and long-term preservation). The risk gene *APOE* (rs429358/rs7412, needed for determining the main ε2, ε3, and ε4 variations) and *PICALM* (rs3851179) alleles were determined with the traditional Sanger sequencing protocol, which is a reliable, standardized DNA sequencing protocol. Genetic data frequencies are shown in Table 2.

**Table 2:** Genetic data frequency.

	Phase I (N = 192)	Phase II (N = 79)
APOE haplotype	Frequency N (%)	
ε2/ε2	1 (0.52%)	0
ε2/ε3	21 (10.9%)	0
ε2/ε4	3 (1.6%)	1 (1.3%)
ε3/ε3	119 (62.0%)	31 (39.2%)
ε3/ε4	46 (24.0%)	45 (57%)
ε4/ε4	2 (1%)	2 (2.5%)
PICALM rs3851179		
A/A	16 (8.3%)	11 (13.9%)
A/G	97 (50.5%)	46 (58.2%)
G/G	79 (41.1%)	22 (27.8%)

### Recording software and devices

**Fig. 3:**
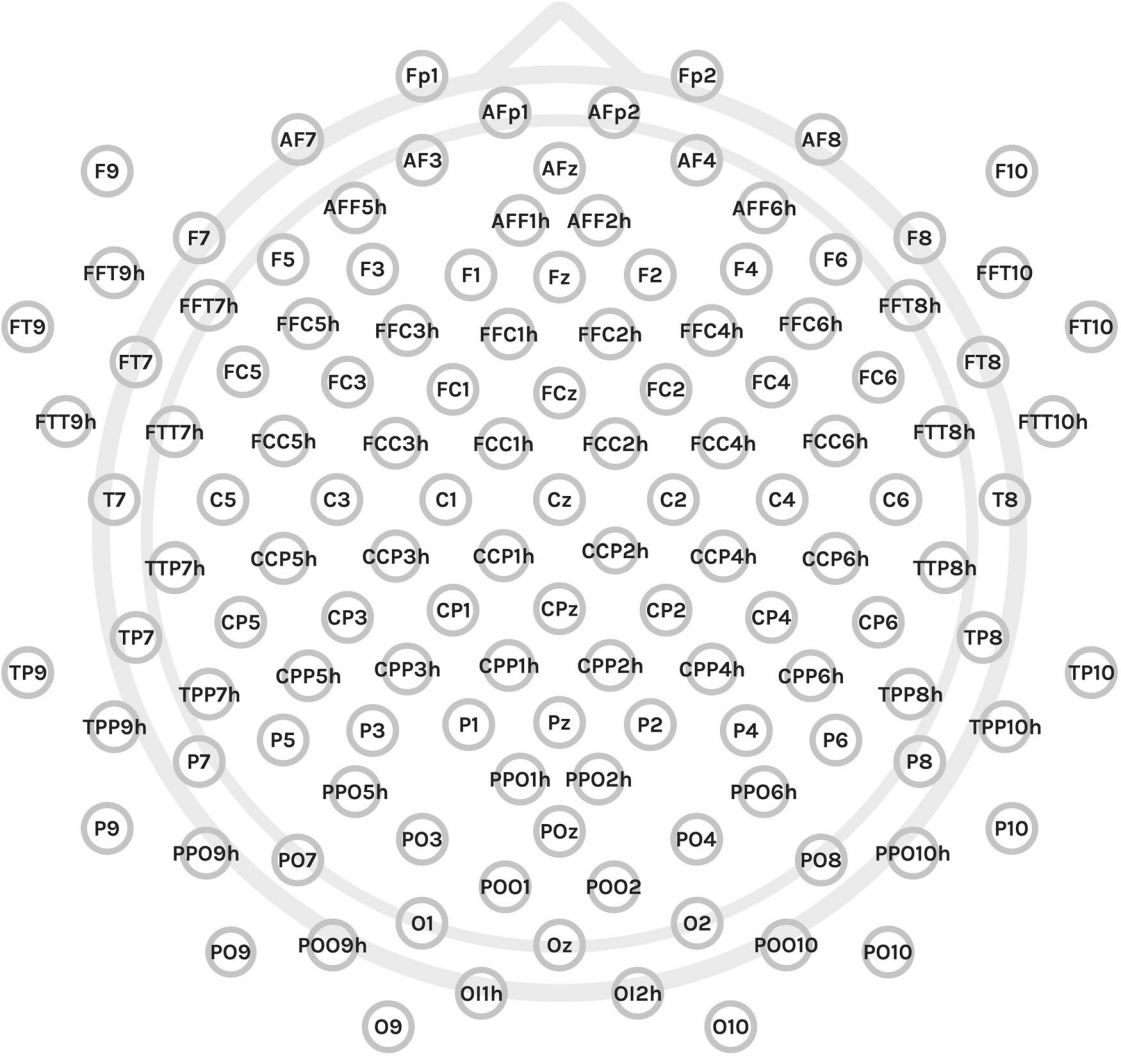
Electrode configuration used for the experiment (128 electrodes).

EEG data was recorded with the use of the Brain Products systems: an actiCHamp amplifier and high-density actiCAP electrode caps with high-montage 128 electrodes included (Brain Products GmbH, Munich, Germany). Standard Brain Products electrode configuration files were used (Fig. 3). The online reference was set at FCz electrode, which can be easily recalculated to any desired off-line reference types, including the average reference. Real electrode localization was obtained with the use of a handheld CapTrak 3D scanner (Brain Products GmbH, Munich, Germany) at the end of each session. The lowest possible impedance was maintained during the recording, on average 5–10 kΩ, by gently rubbing the skin and by EEG gel application. The sampling rate was set to 1,000 Hz. No notch filters or high-pass filters were used during the recording, only the low-pass filter was used and set to 280 Hz.

fMRI experiments were conducted with a 3 T Siemens Prisma FIT scanner (Siemens Medical Systems, Erlangen, Germany). The acquisition parameters were as follows: repetition time (TR)—0.8 s, echo time (TE)—0.038 s, slice thickness—2 mm, and voxel volume—2 × 2 × 2 mm. Images were taken with two-phase encoding direction: anterior-posterior (AP) and posterior-anterior (PA). Additional information on slice timing, echo train length, scanning sequence, variant and other options are included in the database in corresponding.json files and/or corresponding headers of *.nii files (NIfTI-1 Data Format).

### Environment

#### First phase

The participants were recruited through conventional methods (e.g., posters, flyers) and online social media. For both methods, the individuals were directed to a website with information about the study and an online survey where the information about contraindications to participation in the study was indicated. Only subjects with no contraindications were recruited. The list of contraindications was shown to the recruited respondents during the first (psychometric and questionnaire) session and then again before the fMRI session where, again, we asked about MRI-relevant contradictions. The first phase of the study was conducted during individual meetings in a quiet, separate room. At the beginning, the subjects read the information about the study, signed that they had read the document information and signed the informed consent for the study. Then, a session of psychometric testing was conducted; all tests were completed by the subjects on their own, except for the CVLT test, which required researcher involvement. After the testing session, a buccal swab sample was taken for genetic testing (the subjects were instructed on how to take the sample on their own).

#### Second phase

The experiments were conducted in the morning and afternoon, with the possibility for the participant to choose the most convenient time of the session in order to be fully rested. Experimental procedure details are available in Table 3 for EEG and Table 4 for fMRI sessions. The tables include the order of each procedure and task and the estimated time. The high-density cap was prepared before each session to reduce the participant time required during EEG preparation. During the EEG session, the participant was sitting alone in a quiet room on a comfortable chair in front of a monitor. The study was supervised by the researcher sitting in another room via a remote desktop connection to the experimental computer and a LAN camera overlooking the EEG lab.

**Table 3:** Experimental procedure details: EEG session.

No.	Task	Duration time
1.	Filling in the laboratory documents	~5–15 minutes
2.	EEG cap preparation, impedance reduction	~60–80 minutes
3.	Resting state protocol instruction and recording: eyes-open and then eyes-closed condition saved in 1 file	~10 minutes
4.	MSIT instruction and training	~5–8 minutes
5.	MSIT recording	~10 minutes
6.	Sternberg memory task instruction and training	~5–8 minutes
7.	Sternberg memory task recording	~13 minutes
8.	CapTrak session	~30 minutes

**Table 4:** Experimental procedure details: fMRI session.

No.	Task	Duration time
1.	Filling in the laboratory documents and an additional consent for fMRI/MRI study	~5–15 minutes
2.	Participant preparation in the scanner	~5–8 minutes
3.	MSIT instruction and training	~5–8 minutes
4.	Sternberg memory task instruction and training	~5–8 minutes
5,	Resting state protocol instruction	~2–4 minutes
6.	Resting state protocol recording	~15 minutes
7.	MSIT recording	~10 minutes
8.	Sternberg memory task recording	~13 minutes

### Data protection

Data anonymization is a process of removing or altering personally identifiable information from a dataset in such a way that the data can no longer be linked back to an individual. The purpose of anonymization is to protect the privacy of individuals while still allowing the dataset to be used for analysis and research. All data in the database were anonymized with special codes (sub-1, sub-2 etc.), and no direct personal information was maintained through all of the uploaded files. All metadata information related to participants was removed from neuroimaging files.

### Task details

All tasks were written and performed within the Presentation software v. 20.0^50^. Stimuli were displayed in gray color (RGB values: 206, 206, 206) on a dark background (RGB values: 58, 58, 58). The stimuli were displayed on a monitor during the EEG session and on goggles (VisualSystem HD, NNL, nordicneurolabs inc.) during the fMRI session, which also allowed researchers to see participants’ eyes and track their engagement in the task and their wakefulness. Markers used in the EEG files are described in Table 5.

**Table 5:** Markers used in the EEG files for four types of task.

Marker	Task		
	MSIT	Sternberg	Rest
S 1	response ‘1’	Not true	response ‘enter’, after reading subtask instruction
S 2	response ‘2’	True	eyes-open condition start
S 3	response ‘3’	Low-demanding condition	—
S 4	FS condition	High-demanding condition	eyes-closed condition start
S 5	00 condition	—	—
S 10	break	break	eyes-closed instruction
S 11	—	retention	end of the task + sound effect
S 12	—	probe	—

More detailed information about the stimulus is contained in the raw logfiles (see the ‘Data Structure’ paragraph).

Participants responded using a keyboard (Sternberg memory task) and numeric keypad (MSIT) during the EEG session and with a response grip (Smitlab response grip) during the fMRI session. Each file may also contain additional ‘boundary’ type markers that are added automatically by EEGLAB when the file is opened/saved (e.g., at the beginning of each file). In addition, these markers are added where a portion of files are concatenated or deleted. There was a short training session before the main experiment, during which the participants could familiarize themselves with the tasks. The training was repeated if the participants answered incorrectly until they understood the instructions.

#### Multi-source interference task (MSIT)

Multi-source interference task (MSIT) is a cognitive task used to measure adaptive control, cognitive control and cognitive interference^51,52^. The task measures the ability to ignore distracting information and focus on the task-relevant information. Participants are presented with a sequence of three digits (e.g., ‘331’), and they have to identify the target, which is a unique digit, while ignoring distractors (two other, identical digits) by pressing the button corresponding to the digit value, and not its position (in this example ‘1’ would be the correct answer). The digits can be displayed in a congruent way (congruent, low-demanding condition; i.e., the target digit value and its position are the same, which fastens the reaction time towards congruent trials, like ‘122’) or incongruent (incongruent, high-demanding condition; i.e., the target digit location and the response button location are not the same, which prolong reaction times, like ‘331’ trial); this manipulation creates cognitive interference. The MSIT is widely used in cognitive neuroscience research and has been shown to reliably activate the dorsal anterior cingulate cortex, medial prefrontal cortex, and supplementary motor area, among other described areas^53^. It has also been used in clinical populations to assess deficits in various disorders^54,55^ and in healthy aging^56^.

**Fig. 4:**
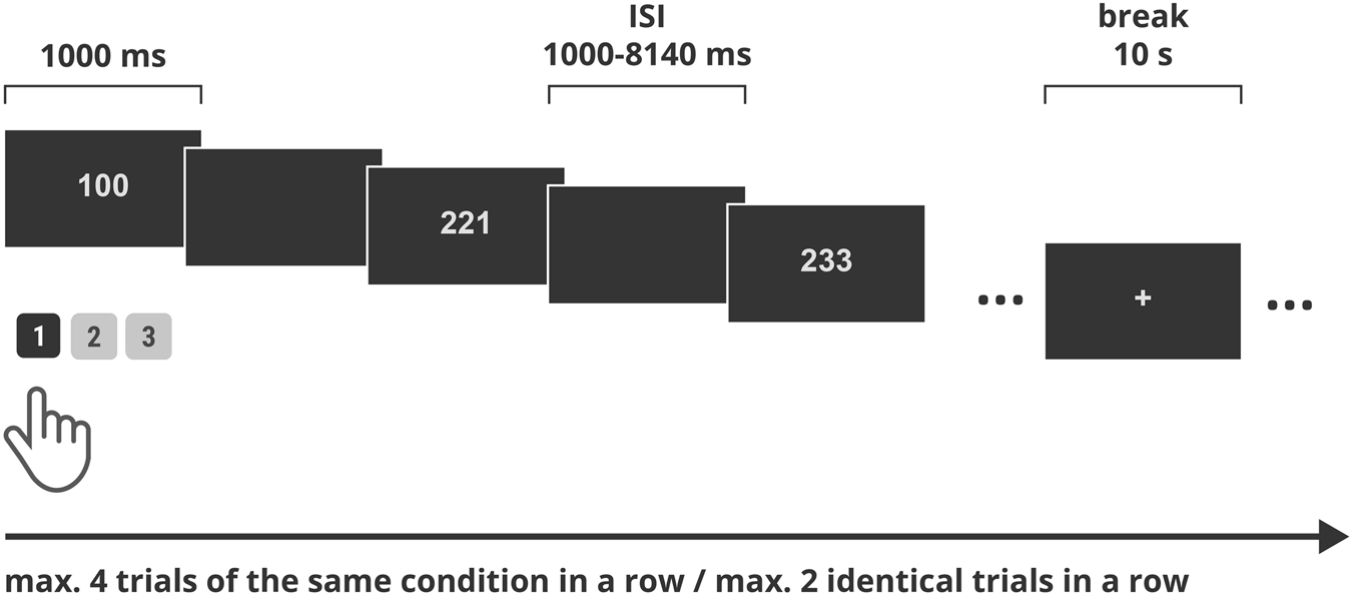
Multi-Source Interference Task design. ISI: interstimulus interval.

Note: During the initial part of the experiment, the MSIT task in fMRI was constructed without the rule of 4 same trials in a row; fMRI files that do not follow the rule: sub_01, sub_20, sub_33, sub_60, sub_78.

Sixteen sets of predefined stimuli sequences were prepared with the use of the OptimizeX genetic algorithm^57^ to reduce the collinearity in the event-related task design, especially for fMRI purposes^58^. Orthogonality of regressors in a general linear model (GLM) of fMRI analysis is needed to reliably estimate the results, as collinearity may impact the estimated contrasts of parameters and, thus, the obtained results^58,59^. Maximally 4 trials of the same condition were displayed in a row, and maximally 2 identical stimuli were displayed in a row. Out of 16 sets of stimuli, both conditions were correlated on a level of 0.14 in 3 designs, 0.16 in 3 designs, 0.17 in 1 design, 0.18 in 7 designs, and 0.19 in 2 obtained designs. The task was prepared in two runs, 83 stimuli per each run which lasted for ~4.2 minutes (with an equalized number of each individual trial, either 13/14 repetitions, therefore the number of trials per condition was also balanced; 166 trials in total; task lasted for ~8.5 minutes in total). The stimuli were displayed on the screen for 1,000 ms, and ISI varied between ~1,000–8,140 ms (on average: ~1,804 ms from all sets). A dark, blank screen was displayed during the interstimulus interval. In each run, a 10-second break, marked with ‘ + ’ was included in the middle of the run. The task design is shown in Fig. 4.

The list of possible trials included:

- Low-demanding condition, congruent: 100, 020, 003.
- High-demanding condition, incongruent: contingency-equalized design presenting each unique stimulus equally often; several parallel subsets of stimuli were used for different sets, either 221, 233, 131 or 212, 332, 311 or 313, 112, 322 or 331, 211, 232. For each individual experiment, a set of incongruent stimuli was randomly drawn to avoid a possible contingency learning bias^60^ that occurs in the original MSIT task. Information about the individual trials was stored in the logfile data (note that leading zeros are not saved; therefore, trial ‘3’ ->‘003’, ‘20’ - > ‘020’ etc.).

#### Sternberg memory task

The Sternberg Memory Task is a widely used psychological paradigm designed to investigate short-term or working memory processes. It was developed by Saul Sternberg in 1966^61^ and has been used extensively in psychological and neuroimaging research. There are various procedures for performing the Sternberg task and studying different memory aspects, but in each instance, a participant is presented with a list of items and then with individual probes. The participants must indicate whether the probe item was part of the original list. Participants are required to respond as quickly as possible and as accurately as possible (without sacrificing accuracy for speed response). In the case of our procedure, the individual was presented with the strings of letters and then with single-letter probes and needed to answer if the letter presented was included in the string (by pressing the right arrow key on the keyboard during the EEG or the first button on the response pad during fMRI), or not (by pressing the left arrow key on the keyboard during the EEG or middle button on the response pad during fMRI).

**Fig. 5:**
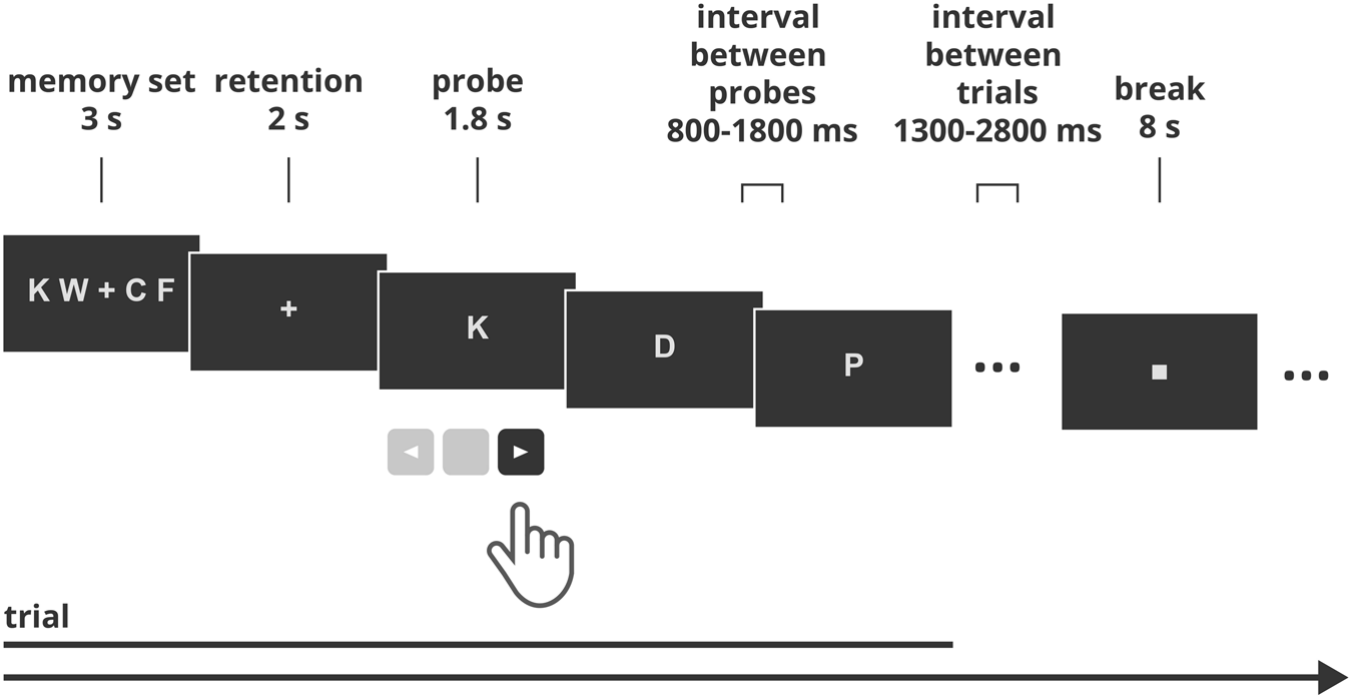
Sternberg's task design. A sample trial of the non-demanding condition is shown. The “K” letter is the target.

Predefined letter combinations, containing only consonants, were used: 24 low-demanding memory sets including 4 letters (e.g., “K W + C F”, “P Z + D J”) and 24 highly-demanding memory sets including 8 letters (e.g., “R T G L + D F B Z”, “W T H J + C F L R”),. Three probes were presented after each memory set, and each included one letter from the consonant set (B, C, D, F, G, H, J, K, L N, P, R, S, T, W, Z). During the retention period, a “ + ” sign was presented on the screen. The interval between the probe presentations was set to the range of 800–1800 ms (in increments of 200 ms, e.g., 800, 1000, 1200, 1400, 1600 or 1800 ms); the interval between the whole trials (understood as a memory set, retention, probes) was set in the range of 1300–2800 ms (increments of 100 ms, e.g., 1300, 1400, 1500 ms etc.). Three breaks lasting for 8 seconds were introduced in the task and were signed by a black square (“▪”). The number of target (letter included in the memory set) and non-target (letter not included in the memory set) probes was balanced across the task. The task design is shown in Fig. 5.

### Information regarding the basic analysis

All statistical analyses were performed using Jeffrey’s Amazing Statistics Program (JASP) (v.0.17.0) (JASP Team, 2023). For quantitative variables (reaction times), a paired t-test was used if the condition of normality of distribution among groups was met (Leven’s test). In case of violation of this condition, a non-parametric Wilcoxon signed-ranks test was used. The boxplots were prepared with Seaborn Python Library.

#### Resting-state session

A resting-state protocol was used to study spontaneous brain activity when no specific task was given. It allows for the study of, among other questions, brain functional connectivity. During the EEG session, subjects were instructed to remain still and quiet, firstly with the eyes open (for 4 minutes) and then with the eyes closed (for 6 minutes). Both conditions were saved in one file. During the fMRI session, a closed-eye condition was used that lasted for 12 minutes.

## Data Records

All of the described data constitutes the Polish Electroencephalography, Alzheimer's Risk-genes, Lifestyle and Neuroimaging (PEARL-Neuro) Database. All the data supporting this article are available as open access data in the OpenNeuro repository^62^.

The database was prepared in Brain Imaging Data Structure (BIDS) standard, a well-recognized and widely-used method of organizing and describing neuroimaging data. The goal of the BIDS standard is to improve the accessibility and reproducibility of neuroimaging data by providing a consistent and easy-to-use format for storing and sharing data. It specifies a set of rules for organizing data into a hierarchy of directories and files and for describing the metadata associated with each dataset. The data for each participant is stored in a dedicated folder identified by the participant code (e.g., ‘sub-01’). Within each participant’s directory, there are separate folders for different types of data, such as functional scans (‘func’), and EEG data (‘eeg’). Inside each of these folders, files are organized according to a standardized naming convention that includes information about the subject, session, modality, and task (for instance, the file ‘sub-01_task-msit_eeg.eeg’ contains EEG data related to the MSIT task for subject number 1). Additionally, event data for EEG and fMRI tasks are stored in tab-separated values (TSV) files with the suffix ‘events.tsv’. In addition to the directory structure, the BIDS standard includes a set of metadata descriptors that provide information about the imaging protocols used to acquire the data, as well as other relevant details such as subject demographics or task descriptions. These metadata are stored in separate files in JSON format, which can be easily read and processed by multiple software tools. Furthermore, the genetic information (accompanied by the ‘genetic_info.json’ file), psychometric results, blood test outcomes, demographic information, family history of dementia information and other participant-related details are stored in the ‘participants.tsv’ table. This table is complemented by a ‘participants.json’ file, which offers a comprehensive description of each column and variables levels within the table.

The rest of the data beyond the BIDS standard is contained in the ‘Sourcedata’ folder, where, for each participant, there is a ‘coords’ folder with raw CapTrak files in.sfp extension (location of the electrodes) and a ‘logfiles’ folder where the raw logfiles are stored with.txt extension.

### Missing data

The full experimental group (N = 200) included 8 more participants (with codes: sub-69, sub-103, sub-111, sub-139, sub-154, sub-157, sub-158 and sub-170), who had not provided the form granting the right to include their data in openly available repository. A few participants withdrew from the experiment or had MRI contradictions for scanning (like metal objects in the body). All fMRI data is missing for subjects: sub-06, sub-22, sub-28, sub-30, sub-50, sub-51, sub-52, sub-61, sub-71; only behavioral fMRI files are missing: sub-77, sub-58; and additionally 28 trials are missing in MSIT task in AP sequence in sub-09 and 4 trials in MSIT AP sequence in sub-24 (only in fMRI.nii.gz files, as raw behavioral files are not missing for these two participants). fMRI data of one participant (sub-60) were excluded from the database due to minor abnormalities on the structural MRI image (with no subjective/objective symptoms, with preserved normal EEG and within normal range scores on neuropsychological tests). For EEG data, only data for Sternberg's task is missing for one participant (sub-51) and data for the resting state due to acquisition and technical problems (sub-55). For CapTrack data, two files are missing: sub-30, sub-51.

## Technical Validation

### Database reliability

The database structure was validated by the BIDS-validator, which confirmed the compliance of the dataset with the BIDS standard. Data are, therefore, correctly formatted and stored. Data are complete, and any missing data are listed in the manuscript. There are no duplicated or dummy entries. The credibility of the database was already confirmed by our reports on genetic differences within the blood profile^17^ and EEG/psychometric traits. We used multiple data collection methods, ensuring data triangulation (neural activity by separate EEG and fMRI sessions, behavioural data or health data including blood test assays, subjective questionnaires and validated psychometric tests). External factors were controlled during both phases of the experiment, as participants took the tests under the same conditions (in the same laboratory rooms, with the same physical conditions).

### Participants profile

The group was equal in terms of gender (89 males, 103 females in the first phase; 39 males, 40 females in the second phase). The participants held mostly higher education (77.6% in the first phase). Basic demographic information is shown in Table 6.

**Table 6:** Demographic data.

Parameter	Phase I (N = 192)		Phase II (N = 79)*	
	Mean ± SD	Median	Mean ± SD	Median
Age (years)	55.10 ± 3.10	55.00	55.30 ± 3.12	56.00
	Frequency N (%)		Frequency N (%)	
Education 1	20 (10.42%)		8 (10.13%)	
Education 2	5 (2.60%)		3 (3.80%)	
Education 3	149 (77.60%)		60 (75.95%)	

*The data are shown for all N = 79 participants from phase II of the experiment, although not all participants underwent all sessions (see the Missing data section). *Valid percent is shown in the table when missing data are excluded from the calculations. Number of participants with missing data: education N = 18/8 (phase I/II). Legend: Education: 1—secondary education, 2—partial higher education, 3—higher education.

The subjects were generally healthy (and described themselves in the same terms), with possible ailments and long-lasting diseases due to age that did not affect cognitive task performance, e.g., hearing or vision problems (corrected), back pain, thyroid problems, allergies and other (information about health are stored in the database, shown in Table 7). Some of the subjects were taking medication on a regular basis (the information about that is included in the database). Mostly, the participants did not have any learning difficulties (83.85% in the first phase).

**Table 7:** Health data.

Parameter	Phase I (N = 192)		Phase II (N = 79)*	
	Mean ± SD	Median	Mean ± SD	Median
BMI	26.88 ± 4.63	25.99	27.40 ± 5.07	26.31
AUDIT	3.80 ± 3.33	3	4.03 ± 2.81	4
	Frequency N (%)		Frequency N (%)	
Learning difficulties 0	8 (4.57%)		4 (5.48%)	
Learning difficulties 1	3 (1.71%)		2 (2.74%)	
Learning difficulties 2	1 (0.57%)		1 (1.37%)	
Learning difficulties 3	0 (0%)		0 (0%)	
Learning difficulties 4	161 (92.00%)		65 (89.04%)	
Allergies 0	—		56 (76.71%)	
Allergies 1	—		17 (23.29%)	
Drugs 0	94 (48.96%)		36 (45.57%)	
Drugs 1	98 (51.04%)		43 (54.43%)	
NSAID 0	48 (25.13%)		21 (26.58%)	
NSAID 1	82 (42.93%)		36 (45.57%)	
NSAID 2	47 (24.61%)		18 (22.79%)	
NSAID 3	11 (5.76%)		2 (2.53%)	
NSAID 4	3 (1.57%)		2 (2.53%)	
Thyroid 0	169 (88.48%)		71 (91.03%)	
Thyroid 1	12 (6.28%)		3 (3.85%)	
Thyroid 2	2 (1.05%)		1 (1.28%)	
Thyroid 3	8 (4.19%)		3 (3.85%)	
Hypertension 0	147 (76.96%)		59 (74.68%)	
Hypertension 1	44 (23.04%)		20 (25.32%)	
Diabetes 0	191 (99.48%)		78 (98.73%)	
Diabetes 1	1 (0.52%)		1 (1.27%)	
Other diseases 0	154 (81.48%)		61 (78.21%)	
Other diseases 1	35 (18.52%)		17 (21.80%)	
Smoking status 0	130 (68.03%)		47 (60.26%)	
Smoking status 1	22 (11.52%)		9 (11.54%)	
Smoking status 2	39 (20.42%)		22 (28.21%)	
Coffee intake 0	18 (9.38%)		5 (6.33%)	
Coffee intake 1	138 (71.88%)		59 (74.68%)	
Coffee intake 2	35 (18.23%)		14 (17.72%)	
Coffee intake 3	1 (0.52%)		1 (1.27%)	
Dementia history 0	130 (67.71%)		50 (63.29%)	
Dementia history 1	58 (30.21%)		28 (35.44%)	
Dementia history 2	4 (2.08%)		1 (1.27%)	

*The data are shown for all N = 79 participants from phase II of the experiment, although not all participants underwent all sessions (see the Missing data section). *Valid percent is shown in the table when missing data are excluded from the calculations. Numbers of participants with missing data: learning difficulties N = 17/6 (phase I/ II), allergies N = 6 (phase II), NSAID (nonsteroidal anti-inflammatory drugs) intake N = 1 (phase I), thyroid diseases N = 1/1 (phase I/II), hypertension N = 1 (phase I), other diseases N = 3/1 (phase I/II), smoking status N = 1/1 (phase I/II). Participants could choose more than one option in regard to the learning deficits question. Legend: Learning difficulties: 0—dyslexia, 1—dysgraphia, 2—dysortography, 3—dyscalculia, 4—none; Allergies: 0—no, 1—yes; Drugs: 0—no, 1—yes; NSAID: 0—none, 1—very rarely (several times a year), 2—rarely (1–4 pills per month), 3—moderately often (5–11 pills per month), 4—often (>12 pills per month); Thyroid diseases: 0—no, 1—hypothyroidism, 2—hyperthyroidism, 3—other; Hypertension: 0—no, 1—yes; Diabetes: 0—no, 1—yes; Other diseases: 0—no, 1—yes; Smoking status: 0—no, 1—yes, 2—in the past; Coffee intake: 0—no, 1—yes, on a daily basis, 2—yes, occasionally, 3—yes, but only decaffeinated coffee. Dementia history: 0—healthy parents, 1—one demented parent, 2—both demented parents.

**Fig. 6:**
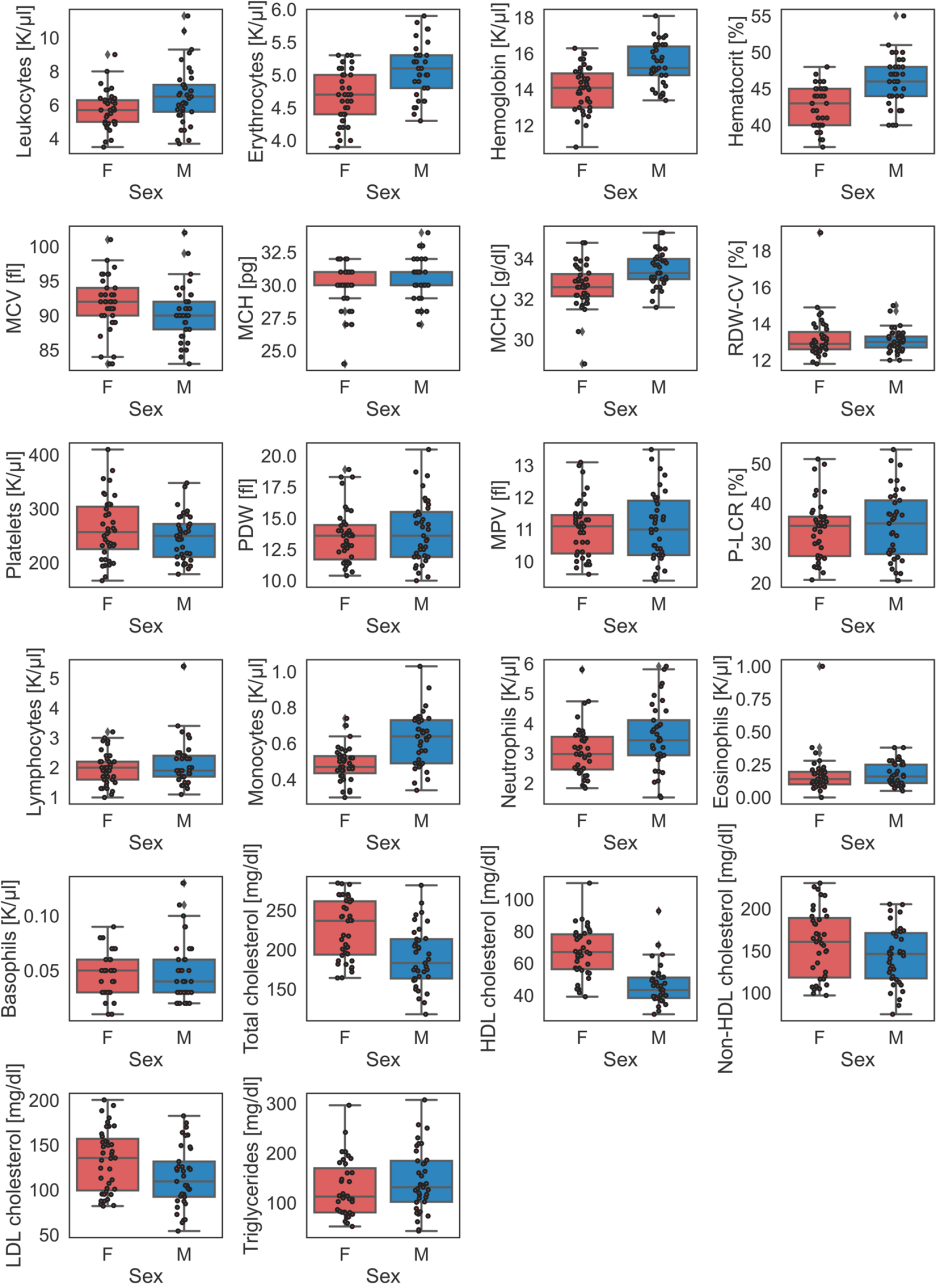
Complete blood count and lipid profile: phase II, N = 77, divided by sex.

Blood results were, on average, normal (Fig. 6), indicating that the group was healthy (although, of course, there are differences in blood results at the participant level). Figure 6 shows the blood test results with the division of females/males, as most blood count parameters differ in regard to sex (Table 8).

**Table 8:** Complete blood count and lipid profile: phase II, N = 77, all participants divided by sex.

Parameter	F/M	Phase II (N = 76; Mean ± SD)	
		M	F
Leukocytes [K/µl]	6.16 ± 1.48	6.57 ± 1.73	5.76 ± 1.10
Erythrocytes [K/µl]	4.87 ± 0.45	5.07 ± 0.40	4.68 ± 0.41
Hemoglobin [K/µl]	14.65 ± 1.38	15.36 ± 1.17	13.97 ± 1.23
Hematocrit [%]	44.21 ± 3.49	45.81 ± 3.38	42.70 ± 2.89
MCV [fl]	91.04 ± 4.05	90.32 ± 3.94	91.72 ± 4.09
MCH [pg]	30.13 ± 1.64	30.32 ± 1.56	29.95 ± 1.70
MCHC [g/dl]	33.06 ± 1.08	33.50 ± 0.86	32.63 ± 1.11
RDW-CV [%]	13.15 ± 0.96	13.08 ± 0.64	13.21 ± 1.20
Platelets [K/µl]	255.67 ± 50.93	247.70 ± 42.43	263.23 ± 57.38
PDW [fl]	13.75 ± 2.34	13.95 ± 2.53	13.57 ± 2.16
MPV [fl]	11.07 ± 0.98	11.10 ± 1.08	11.03 ± 0.90
P-LCR [%]	33.93 ± 8.18	34.34 ± 8.92	33.55 ± 7.49
Lymphocytes [K/µl]	2.05 ± 0.70	2.14 ± 0.78	1.96 ± 0.54
Monocytes [K/µl]	0.55 ± 0.14	0.62 ± 0.15	0.49 ± 0.09
Neutrophils [K/µl]	3.31 ± 1.00	3.56 ± 1.10	3.10 ± 0.85
Eosinophils [K/µl]	0.18 ± 0.13	0.18 ± 0.09	0.17 ± 0.16
Basophils [K/µl]	0.05 ± 0.02	0.05 ± 0.03	0.05 ± 0.02
Total cholesterol [mg/dl]	208.29 ± 41.79	189.92 ± 38.01	225.81 ± 37.83
HDL cholesterol [mg/dl]	57.07 ± 17.40	46.41 ± 12.43	67.04 ± 15.17
Non-HDL cholesterol [mg/dl]	151.21 ± 37.96	143.42 ± 34.19	158.62 ± 40.26
LDL cholesterol [mg/dl]	123.97 ± 35.40	114.39 ± 33.71	133.06 ± 34.96
Triglycerides [mg/dl]	136.23 ± 59.98	145.14 ± 62.54	127.78 ± 56.96

### Psychometric results

**Fig. 7:**
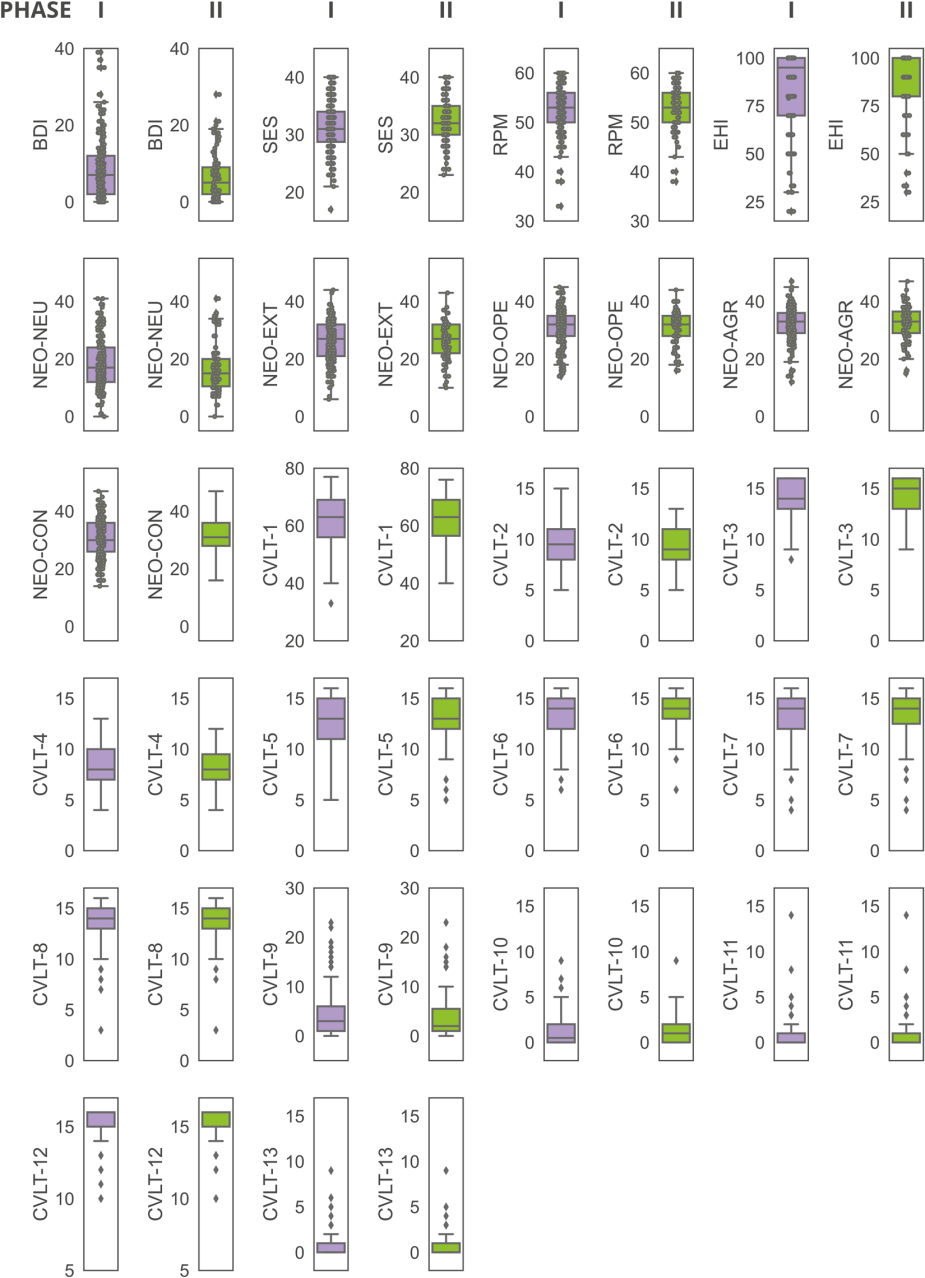
Psychometric test results: BDI, SES, RPM, EHI, NEO and CVLT for both study phases.

Participants described themselves as right-handed, and the EHI test result confirmed right-handedness (Table 9, Fig. 7). The average scores of BDI-II and SES were within the normal range for a healthy population, but in each test, there were participants with higher/lower scores than the mean. In the case of the intelligence test (RPM), respondents scored high (although it was a simple, classic version of the test). Similarly, in the case of the memory test (CVLT), participants scored, on average, very well. Table 9 also shows the average scores for the Big Five personality traits measured by the NEO-FFI test. We included the scores for 14 main scales of the Mini-Cope questionnaire in Table 9, without any further division, as those may be different for different study purposes and techniques, and usually, such an additional aggregation of scales is prepared for different scientific purposes^41^.

**Table 9:** Psychometric data.

Parameter	Phase I (N = 192)		Phase II (N = 79)	
	Mean ± SD	Median	Mean ± SD	Median
BDI	6.33 ± 6.00	5	6.00 ± 5.77	6
SES	32.24 ± 4.05	32	32.78 ± 4.28	33
RPM	52.98 ± 4.61	53	52.78 ± 4.97	53
EHI	88.19 ± 19.04	100	88.33 ± 20.13	100
NEO-NEU	16.38 ± 8.11	15	16.18 ± 8.04	13.5
NEO-EXT	26.54 ± 6.85	27	27.80 ± 7.07	28.5
NEO-OPE	31.08 ± 5.63	32	31.23 ± 5.12	32
NEO-AGR	32.48 ± 6.22	33	33.53 ± 6.64	35
NEO-CON	31.13 ± 6.93	31	32.30 ± 7.25	32.5
CVLT-1	61.83 ± 9.22	63	61.91 ± 8.60	63
CVLT-2	9.51 ± 2.02	9.5	9.42 ± 1.82	9
CVLT-3	13.89 ± 2.07	14	14.11 ± 1.87	15
CVLT-4	8.26 ± 2.03	8	8.20 ± 2.02	8
CVLT-5	12.75 ± 2.70	13	12.95 ± 2.65	13
CVLT-6	13.44 ± 1.98	14	13.51 ± 1.91	14
CVLT-7	13.42 ± 2.53	14	13.54 ± 2.64	14
CVLT-8	13.64 ± 2.04	14	13.68 ± 2.22	14
CVLT-9	4.10 ± 4.50	3	4.10 ± 4.87	2
CVLT-10	1.14 ± 1.64	0.5	1.14 ± 1.64	1
CVLT-11	0.75 ± 1.53	0	1.11 ± 2.08	0
CVLT-12	15.38 ± 1.06	16	15.37 ± 1.07	16
CVLT-13	0.52 ± 1.11	0	0.63 ± 1.40	0
MC-1	2.37 ± 0.56	2.5	2.39 ± 0.56	2.50
MC-2	2.42 ± 0.56	2.5	2.32 ± 0.57	2.50
MC-3	1.86 ± 0.68	2.0	1.88 ± 0.60	2.00
MC-4	2.13 ± 0.64	2.0	2.12 ± 0.62	2.00
MC-5	0.90 ± 0.62	1.0	0.96 ± 0.65	1.00
MC-6	0.74 ± 0.98	0.00	0.68 ± 0.98	0.00
MC-7	1.72 ± 0.72	2.00	1.72 ± 0.75	2.00
MC-8	1.68 ± 0.65	2.00	1.55 ± 0.66	1.50
MC-9	1.58 ± 0.71	1.5	1.66 ± 0.63	2.00
MC-10	0.38 ± 0.53	0.00	0.40 ± 0.55	0.00
MC-11	1.24 ± 0.58	1.5	1.28 ± 0.56	1.50
MC-12	0.30 ± 0.51	0.00	0.32 ± 0.53	0.00
MC-13	0.70 ± 0.58	0.50	0.74 ± 0.57	1.00
MC-14	1.18 ± 0.76	1.00	1.10 ± 0.70	1.00

*The data are shown for all N = 79 participants from phase II of the experiment, although not all participants underwent all sessions (see the Missing data section). *Mean ± standard deviation and median data are shown in the table. Numbers of participants with missing data: NEO N = 1 (phase I). Legend: NEO-NEU: neuroticism scale; NEO-EXT: extraversion scale; NEO-OPE: openness scale; NEO-AGR: agreeableness scale; NEO-CON: conscientiousness scale; MC: Mini-Cope questionnaire.

### Quality control for neuroimaging data

EEG data were visually inspected, and the acceptable quality of the obtained EEG signals was confirmed. The mean level of impedance obtained during each EEG study is described in Supplementary Table 1, which confirms high standards of EEG acquisition.

### Tasks reliability

**Fig. 8:**
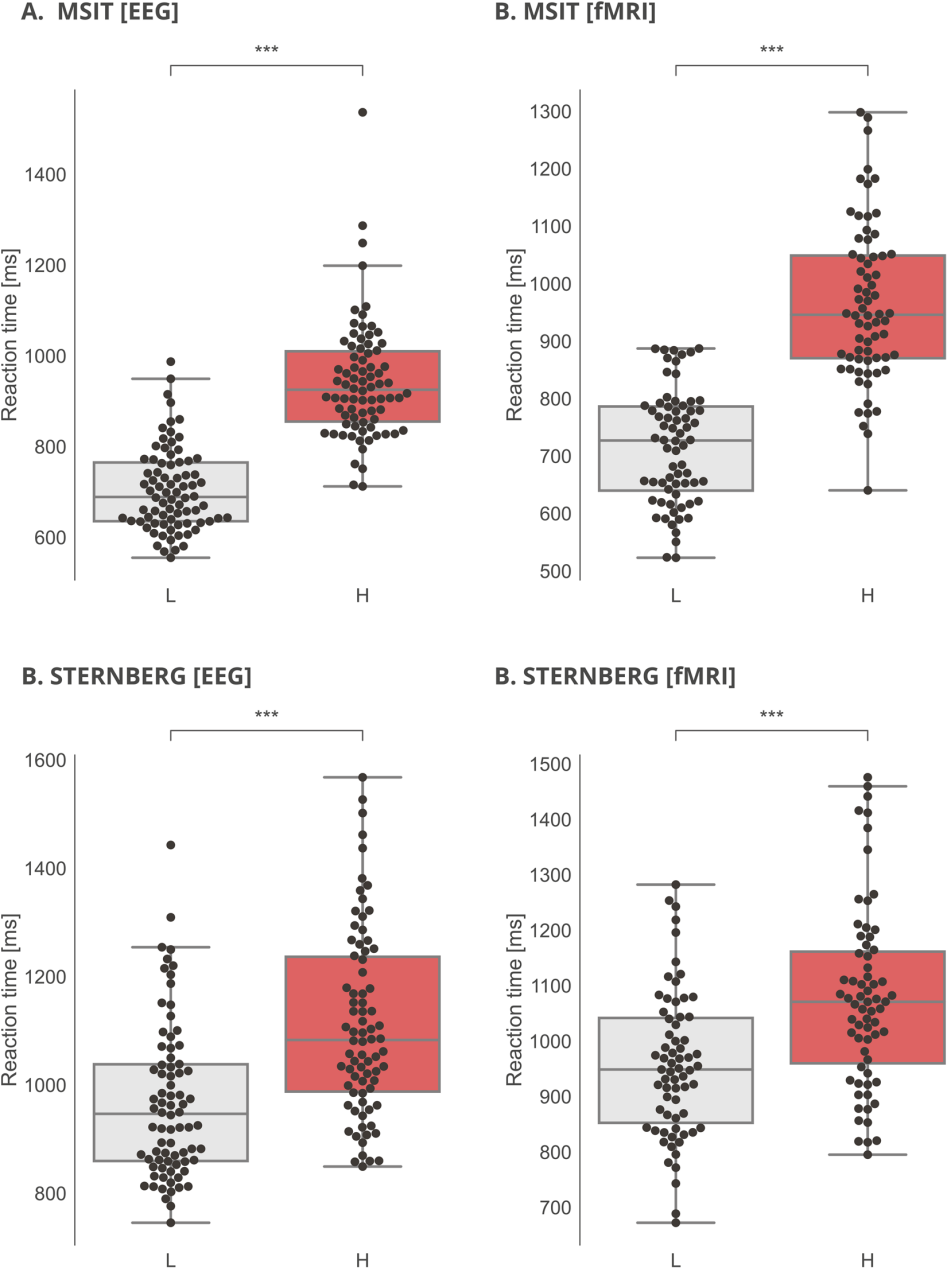
Main effects of task complexity in both the MSIT and Sternberg Memory Task during EEG and fMRI sessions. *** < 0.001. Figure note: L—low-demanding (00) condition; H—high-demanding (FS) condition.

The behavioral results from the selected tasks confirmed data from other previously published experiments. The behavioral data revealed a gradual increase in reaction times in more demanding conditions, which validated the chosen paradigms (Table 10, Fig. 8). Participants were significantly slower to respond to the high-demanding incongruent MSIT condition versus the low-demanding congruent condition in EEG (Wilcoxon Signed-Ranks Tests, as the normality assumption measured by Shapiro-Wilk was violated, z = 7.67, p < 0.001) and in fMRI (Wilcoxon Signed-Ranks Tests, as the normality assumption measured by Shapiro-Wilk was violated, z = 7.17, p < 0.001). Similarly, participants were significantly slower to respond to a highly-demanding Sternberg condition versus a low-demanding condition in EEG (paired t-test, t(77) = 15.32, p < 0.001) and in fMRI (paired t-test, t(66) = 11.63, p < 0.001).

**Table 10:** Reaction time statistics for MSIT and Sternberg Memory Task.

	EEG		fMRI	
	Mean ± SD	Median	Mean ± SD	Median
MSIT L (00)	705.49 ± 92.09	688.84	716.05 ± 98.55	727.12
MSIT H (FS)	942.58 ± 129.13	925.21	963.69 ± 137.02	945.87
Sternberg L	968.65 ± 143.07	946.62	956.19 ± 131.66	948.82
Sternberg H	1111.80 ± 171.91	1082.99	1077.27 ± 164.14	1070.92

*L—low demanding condition (MSIT congruent, 00), H—high demanding condition (MSIT incongruent, FS).

## Usage Notes

There are certain limitations of this dataset, as stated below:

- The demographic/health questionnaire and some of the psychometric tests used (like BDI-II, SES etc.) are self-report tests, for which there is a potential for social desirability bias, response bias, or recall bias.
- Blood testing was conducted only in the second stage of the study, and other databases with much larger sample sizes and genetic screening for *APOE* gene already exist.
- The database do not include information about well-established AD biomarkers, such as levels of for example plasma derived amyloid-β 42/40, or phosphorylated tau.

### Supplementary information

Supplementary Table 1

## Data Availability

No custom code was used. We share raw data.
