# Supplementary material for: PEARL-Neuro Database: EEG, fMRI, health and lifestyle data of middle-aged people at risk of dementia
Source: Sci Data. 2024 Mar 7;11:276. doi: 10.1038/s41597-024-03106-5 (PMC10920678; doi:10.1038/s41597-024-03106-5)
Supplement: Supplementary file 1 — Supplementary Table 1 [file 41597_2024_3106_MOESM1_ESM.docx]

Supplementary Table 1. Mean impedance levels for each task and each participant in the EEG study

| Subject ID | Mean impedance level [kΩ] | | |
| --- | --- | --- | --- |
|  | REST | MSIT | Sternberg |
| sub-01 | 6.88 | 6.88 | 6.88 |
| sub-02 | 5.02 | 4.96 | 5.04 |
| sub-03 | 7.45 | 6.52 | 5.20 |
| sub-04 | 8.16 | 8.16 | 8.16 |
| sub-05 | 6.19 | 5.73 | 4.87 |
| sub-06 | 8.49 | 7.92 | 7.92 |
| sub-07 | 11.54 | 10.01 | 9.52 |
| sub-08 | 7.10 | 5.23 | 4.28 |
| sub-09 | 9.23 | 9.23 | 8.10 |
| sub-10 | 4.75 | 4.46 | 4.28 |
| sub-11 | 4.48 | 3.91 | 3.91 |
| sub-12 | 13.69 | 13.01 | 12.23 |
| sub-13 | 7.47 | 6.81 | 6.39 |
| sub-14 | 8.44 | 8.44 | 6.53 |
| sub-15 | 7.56 | 7.56 | 5.23 |
| sub-16 | 5.54 | 5.01 | 4.85 |
| sub-17 | 8.49 | 8.49 | 8.49 |
| sub-18 | 8.89 | 8.89 | 7.90 |
| sub-19 | 7.73 | 6.83 | 6.83 |
| sub-20 | 6.97 | 6.97 | 6.97 |
| sub-21 | 6.47 | 6.47 | 6.47 |
| sub-22 | 3.73 | 3.37 | 3.37 |
| sub-23 | 1.86 | 1.76 | 1.85 |
| sub-24 | 15.69 | 14.50 | 13.38 |
| sub-25 | 12.35 | 11.76 | 11.76 |
| sub-26 | 5.92 | 5.12 | 4.69 |
| sub-27 | 6.25 | 6.25 | 5.08 |
| sub-28 | 7.25 | 6.37 | 5.83 |
| sub-29 | 4.77 | 4.77 | 3.17 |
| sub-30 | 3.65 | 2.87 | n/a |
| sub-31 | 6.23 | 5.53 | 5.08 |
| sub-32 | 5.78 | 4.23 | 3.60 |
| sub-33 | 6.01 | 6.01 | 6.01 |
| sub-34 | 5.03 | 5.03 | n/a |
| sub-35 | 6.06 | 4.30 | 3.64 |
| sub-36 | 11.62 | 10.37 | 10.37 |
| sub-37 | 5.52 | 7.05 | 5.31 |
| sub-38 | 4.67 | 4.04 | 3.93 |
| sub-39 | 6.18 | 5.48 | 5.48 |
| sub-40 | 7.54 | 6.60 | 5.70 |
| sub-41 | 9.10 | 8.27 | 7.65 |
| sub-42 | 19.19 | 19.19 | 16.45 |
| sub-43 | 8.83 | 8.56 | 8.56 |
| sub-44 | 2.19 | 2.36 | 2.36 |
| sub-45 | 9.40 | 9.40 | 9.50 |
| sub-46 | 3.68 | 3.12 | 2.88 |
| sub-47 | 7.33 | 7.14 | 6.96 |
| sub-48 | 10.12 | 10.12 | 10.20 |
| sub-49 | 6.62 | 5.75 | 5.31 |
| sub-50 | 6.44 | 6.16 | 6.16 |
| sub-51 | 11.97 | 15.19 | n/a |
| sub-52 | 12.56 | 10.59 | 10.59 |
| sub-53 | 5.33 | 5.18 | 5.18 |
| sub-54 | 12.38 | 12.44 | 12.19 |
| sub-55 | n/a | 5.10 | 5.00 |
| sub-56 | 6.64 | 7.09 | 7.09 |
| sub-57 | 6.06 | 6.06 | 6.06 |
| sub-58 | 10.30 | 10.30 | 7.85 |
| sub-59 | 9.52 | 8.56 | 8.56 |
| sub-60 | 5.54 | 5.54 | 5.54 |
| sub-61 | 9.79 | 9.42 | 8.81 |
| sub-62 | 9.34 | 7.94 | 6.98 |
| sub-63 | 12.76 | 11.63 | 10.86 |
| sub-64 | 5.81 | 5.81 | 5.10 |
| sub-65 | 7.67 | 6.88 | 6.88 |
| sub-66 | 6.79 | 6.12 | 5.99 |
| sub-67 | 3.60 | 3.05 | 2.94 |
| sub-68 | 6.79 | 5.55 | 5.23 |
| sub-70 | 12.24 | 9.55 | n/a |
| sub-71 | 10.21 | 9.94 | 9.94 |
| sub-72 | 8.02 | 7.59 | 7.19 |
| sub-73 | 7.27 | 7.27 | 5.88 |
| sub-74 | 15.90 | 14.40 | 14.40 |
| sub-75 | 5.08 | 4.25 | 3.75 |
| sub-76 | 11.46 | 10.21 | 8.59 |
| sub-77 | 4.92 | 4.92 | 4.63 |
| sub-78 | 6.00 | 6.06 | 6.42 |
| sub-79 | 5.40 | 5.40 | 4.92 |
| sub-80 | 8.34 | 7.79 | 7.79 |
